# Context-Based e2e Autonomous Operation in B5G Networks

**DOI:** 10.3390/s24051625

**Published:** 2024-03-01

**Authors:** Shaoxuan Wang, Marc Ruiz, Luis Velasco

**Affiliations:** Advanced Broadband Communications Center (CCABA), Universitat Politècnica de Catalunya (UPC), 08034 Barcelona, Spainluis.velasco@upc.edu (L.V.)

**Keywords:** beyond 5G networks, autonomous operation, context sharing, traffic prediction

## Abstract

The research and innovation related to fifth-generation (5G) networks that has been carried out in recent years has decided on the fundamentals of the smart slice in radio access networks (RANs), as well as the autonomous fixed network operation. One of the most challenging objectives of beyond 5G (B5G) and sixth-generation (6G) networks is the deployment of mechanisms that enable smart end-to-end (e2e) network operation, which is required for the achievement of the stringent service requirements of the envisioned use cases to be supported in the short term. Therefore, smart actions, such as dynamic capacity allocation, flexible functional split, and dynamic slice management need to be performed in tight coordination with the autonomous capacity management of the fixed transport network infrastructure. Otherwise, the benefits of smart slice operation (i.e., cost and energy savings while ensuring per-slice service requirements) might be cancelled due to uncoordinated autonomous fixed network operation. Notably, the transport network in charge of supporting slices from the user equipment (UE) to the core expands across access and metro fixed networks. The required coordination needs to be performed while keeping the privacy of the radio and fixed network domains, which is important in multi-tenant scenarios where both network segments are managed by different operators. In this paper, we propose a novel approach that explores the concept of context-aware network operation, where the slice control anticipates the aggregated and anonymized information of the expected slice operation that is sent to the fixed network orchestrator in an asynchronous way. The context is then used as the input for the artificial intelligence (AI)-based models used by the fixed network control for the predictive capacity management of optical connections in support of RAN slices. This context-aware network operation aims at enabling accurate and reliable autonomous fixed network operation under extremely dynamic traffic originated by smart RAN operation. The exhaustive numerical results show that slice context availability improves the benchmarking fixed network predictive methods (90% reduction in prediction maximum error) remarkably in the foreseen B5G scenarios, for both access and metro segments and in heterogeneous service demand scenarios. Moreover, context-aware network operation enables robust and efficient operation of optical networks in support of dense RAN cells (>32 base stations per cell), while the benchmarking methods fail to guarantee different operational objectives.

## 1. Introduction

Future radio access networks (RANs) will extend the current fifth-generation (5G) technologies and will operate with massive and heterogeneous small-cell deployments in support of diverse beyond 5G (B5G) and sixth-generation (6G) use cases, demanding high bandwidth and stringent latency requirements [1]. In order to cope with such demand, RAN cells need to be planned with a high number of base stations (BSs) per cell; which leads to overprovisioning and high energy consumption. To reduce both, the operational mode (active–sleep) of BSs can be dynamically managed as a function of the current user equipment (UE) traffic requirements [2].

Thanks to RAN and 5G core *virtualization*, functional splits [3] can be used to distribute the signal processing chain between a distributed unit (DU) and a centralized unit (CU) in the RAN and the user plane function (UPF) in the core, which can be deployed at different sites of the network [4]. The adoption of *flexible function split* is a promising solution that allows the dynamic adaptation to different quality of service (QoS) requirements, which substantially improves RAN efficiency [5]. Recently, open radio initiatives, such as O-RAN [6], have allowed the deployment and management of *slices* to exploit the aforementioned dynamicity and adaptability capabilities, as well as the achievement of a virtualized, interoperable RAN among multiple vendors [7]. Hence, *smart slice operation* can be achieved by combining dynamic RAN resource allocation and slice management with flexible functional split management [8], which in turn can be extended with advanced artificial intelligence (AI) capabilities for the QoS assurance of stringent services [9].

The deployment of multilayer optical networks in access and metro segments plays a fundamental role in meeting the end-to-end (e2e) requirements of slices [10]. Several recent works have focused on the 5G and B5G RAN provisioning that is supported by the resources (computation and connectivity) provided by the underlying access and metro networks. For instance, the authors in [11] tackled the problem of DU/CU placement in the access and metro networks for power consumption minimization that are subject to functional split, latency, and capacity requirements. Targeting more advanced B5G scenarios, the authors in [12] considered functional split, traffic split, different placement options for virtual functions, and network slice-specific requirements in a joint provisioning problem. Moreover, the problem of combining DU/CU placement with connection provisioning in underlying optical networks was presented in [13] and solved for different types of services. Although those contributions present valid performance evaluation studies, they do not focus on the practical integration of smart RAN and fixed transport networks, which is a major challenge to be tackled to guarantee reliable and efficient communication networks in support of B5G and 6G services [14].

As with smart slice operation, *autonomous network operation* is required to allow fixed (optical) networks to operate efficiently. Such an operational paradigm is typically based on autonomous control loops, where monitoring data are continuously gathered and analyzed by means of AI models and algorithms that trigger the actions to be performed in the network, e.g., the adaptation of optical capacity to the current and expected traffic demand. This is particularly interesting when the digital sub-carrier multiplexing (DSCM) technology is used, as sub-carriers can be activated and deactivated in near real time, which leads to operational benefits, including energy savings [15]. Note that this AI-based operation can be built on top of a distributed architecture that enables local control loops and efficient resource management without saturating the centralized systems in charge of, e.g., e2e service provisioning [16].

Dealing with highly variable traffic is the main challenge for autonomous transport network operation; this traffic also becomes unpredictable as a consequence of smart slice operation. In a classical 4G scenario, the traffic injected by RAN cells into the fixed network typically fluctuates with smooth patterns that are highly correlated with UE demand [17]. However, depending on the functional split and DU/CU virtual function placement, B5G slices carry a mix of front-haul (F-H), mid-haul (M-H), and back-haul (B-H) traffic that depends not only on UE demand, but also on slice operation. Thus, actions such as activating a new BS and changing the functional split or the placement of virtual functions introduce large and sudden changes in the traffic of slices and, consequently, in the underlying optical connections supporting them [18].

In view of the above, it is clear that smart slice operation and autonomous fixed network operation must be coordinated to achieve the required e2e performance. It is worth noting that per-slice coordination has recently been proposed, e.g., to guarantee QoS target performance by exchanging service-related parameters [19]. This approach is valid only for restricted scenarios where the RAN operation is fixed and both the RAN and the fixed network domains belong to one single operator; thus, detailed information from UE activity can be shared from one domain to the other. However, when autonomous fixed network operation deals with a mix of several slices from different tenants, as well as other fixed access flows, information sharing among the domains deserves dedicated consideration to avoid the revealing of internal domain details. Indeed, sharing aggregated data and/or models allows the preservation of privacy while keeping the value of transferred knowledge [20].

Recently, some initiatives have explored the use of contextual information to improve the use of RAN resources in B5G scenarios by sharing data from the UE to the RAN control in an asynchronous and private way [21]. Inspired by that concept, in this paper we extend the coordination between RAN and fixed networks in [19] and the knowledge management in [20] and present context-aware autonomous network operation that enables e2e smart operation by combining the operation of slices and the fixed network in an effective and privacy-preserving way. The main objective of the proposed context-aware operation is to improve the current autonomous fixed network operation approaches [15] that fail to guarantee acceptable QoS assurance under extremely dynamic traffic originated by smart RAN operation. The key concept is the definition of context variables that are passed from the slice manager to the software-defined networking (SDN) control performing dynamic capacity resource allocation in the fixed transport network. Context variables contain relevant information about the configuration of the slices in an aggregated way so as to preserve the privacy of individual services and the UE. Moreover, the context is updated asynchronously, e.g., before a significant slice reconfiguration is performed. In this way, the volume of data exchanged between domains is minimized, as is the frequency of the exchange. Specifically, the contributions of this paper are presented and organized in the following sections:

- Section 2 provides an overview of the e2e B5G/6G reference high-level architecture and topology, including RAN, 5G core, and fixed transport segments, as well as the architecture considered in this work. This extends the topology and architecture in [22] for B5G fixed networks with the inclusion of the B5G/6G RAN and core segment.
- Section 3 introduces the main concepts and actions of smart slice operation, highlighting the impact of slice management actions on the fixed transport operation and setting up the main challenges and requirements of contextual information sharing.
- Section 4 presents the context-aware autonomous network operation procedure. To the best of our knowledge, this is the first work using contextual information sharing between slice management and fixed transport domains for an autonomous e2e network operation that coordinates and encompasses both the RAN and fixed network operation. The section presents the main algorithms and models involved, which include asynchronous context updates and context-aware AI-based capacity reconfiguration modules.

Section 5 presents numerical results to illustrate the benefits of the proposed context-aware autonomous network operation compared to benchmarking approaches, where slice management and the fixed network operate independently. Finally, Section 6 concludes the paper. Table 1 summarizes the basic topics of our contribution and compares them with the contributions of the already mentioned works in the literature. As can be observed, our contribution is the one that covers all the highlighted topics.

## 2. B5G Reference Scenario

In the RAN, we assume that a cell consists of a single macro BS (MBS) and a number of micro BSs (µBSs). MBSs provide full coverage within their cells and provide the minimum capacity to absorb users’ traffic, whereas µBSs complement the capacity of the MBS within a limited area of the cell. We assume that µBSs provide two operational modes: (*i*) *active*, where the µBS is switched on and fully operational, and (*ii*) *sleep*, where the µBS is switched off. Without loss of generality, we assume that the radio units (RUs) on both the MBS and the µBSs provide support for e2e traffic flows. RAN cells provide radio connectivity to the UE and require one of the following main service classes [23]: (*i*) enhanced mobile broadband (eMBB), (*ii*) ultra-reliable low-latency communications (URLLC), or (*iii*) massive Internet of Things (mIoT). It is worth mentioning that eMBB typically requires a large capacity (~150 Mb/s per UE and service) with relaxed e2e latency requirements (~4 ms from the UE to the core). Conversely, the URLLC service has very stringent latency requirements (~1 ms) and reduced capacity. Finally, mIoT is typically highly distributed, which entails the management of a large amount of UE injecting moderated bandwidth (in the order of tens of Mb/s) with intermediate target e2e latency assurance (~2 ms).

**Figure 1:**
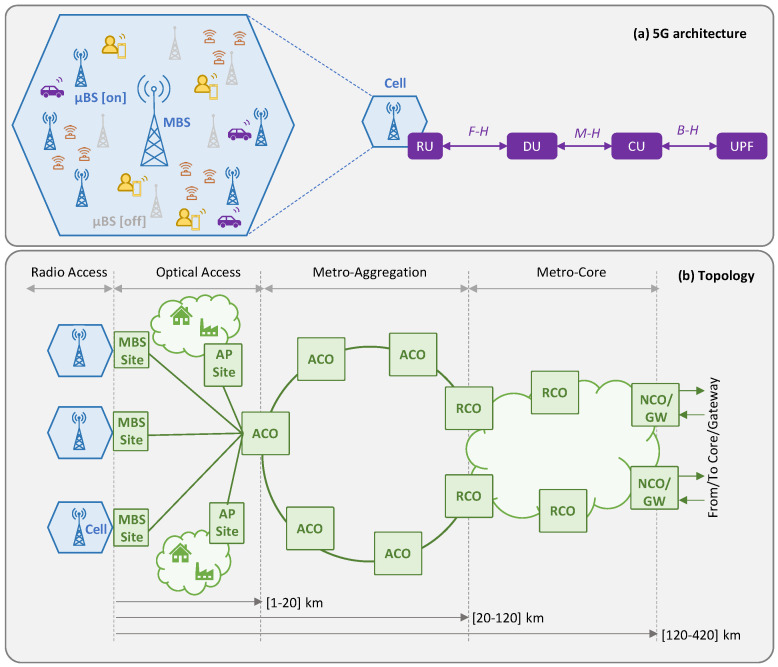
Reference 5G architecture (**a**) and topology (**b**).

Figure 1a illustrates the 5G high-level reference architecture considered in this work, where the traffic generated by the UE in a cell sequentially traverses a number of functions, namely RU, DU, and CU, until reaching the UPF that serves as a breakout point of the 5G core [24]. Thus, the resultant *graph* can be split into four different *slice links*, characterized by the RAN segment, i.e., radio (between UE and RU), F-H (between RU and DU), M-H (between DU and CU), and B-H (between CU and UPF). All these functions can be virtualized and run on the computing resources (servers, virtual machines, or containers) that are available at the different sites of the network. The B5G architecture is supported by resources in the fixed network infrastructure, for both connectivity, i.e., capacity and ensured latency, and computing. The e2e B5G reference topology considered in this work is depicted in Figure 1b, where the main network segments connecting the sites and the central offices (COs) are sketched. This topology is based on the reference high-level topology from the major European network operators presented in [22]. Therefore, the traffic of a cell enters the fixed network. Specifically, an access optical network connects the cell sites with their reference *access CO* (ACO). Typically, the distances between the RAN cells and their ACO site are short, i.e., from a few to tens of km. In addition to their optical transport and switching capabilities, ACO sites have small datacenters equipped with computing and storage resources that enable the deployment of virtualized DU/CU functions, as well as other UPF functions. Typically, ACOs aggregate traffic from various RAN cells in the nearby area, as well as from other access technologies, such as residential gateways or customer edge premises. ACOs are interconnected with each other and with *regional COs* (RCO) by metro aggregation networks. RCOs are further from the UE (around hundreds of km) and larger and more complex than ACOs; hence, they can host more virtualized functions and achieve higher efficiency. Finally, RCOs are interconnected with *national COs* (NCO) by means of a meshed metro core network, which provides large computational capabilities and serves as a gateway to other networks.

**Figure 2:**
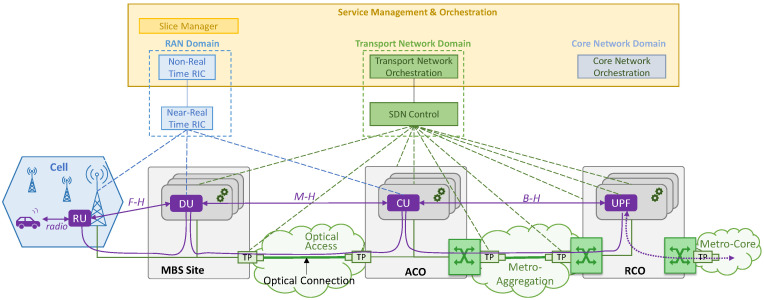
High-level architecture.

Figure 2 illustrates the overall architecture considered in this work, including the control and orchestration planes; this architecture is an adapted version of the O-RAN architecture [6]. The main entity responsible for RAN domain management is the RAN intelligent controller (RIC) that is in charge of a wide set of actions, such as QoS-based resource optimization, traffic steering, and RAN energy efficiency, just to mention a few. The RIC is divided into the near real-time RIC and non-real-time RIC. The near real-time RIC controls the RAN elements and their resources by means of local control loops that typically run in the range of 10 ms to 1 s; it receives policies from the non-real-time RIC that runs in the service management and orchestration system, which enables wide control loops that require an execution time of more than 1 s. For the sake of simplicity, we hereafter refer to the unified RAN control entity that combines near real-time and non-real-time operation as simply the RIC. Specifically, we assume that the RIC deals with cell configuration, e.g., BS on/off switching, and that it manages the DU/CU placement for each slice [7]. The core network orchestrator is responsible for the core functions; specifically, we assume that it manages the UPF placement for each slice. A slice manager is in charge of making decisions related to the configuration of each slice for service level agreement assurance. Finally, in the transport network domain, the orchestrator coordinates actions with the SDN control plane. It is worth noting that the orchestrator layer provides O-Cloud functionality [6], i.e., it manages the computing nodes running in each site, as well as the connectivity between sites [25].

Without loss of generality and in line with [22], the sites are equipped with optical transponders (TP) that allow their connection to remote sites by establishing an optical connection. In addition, in this paper we consider DSCM TPs, which can allocate a variable number of sub-carriers to adapt the capacity to the traffic needs.

The mapping of the slice links connecting functions onto optical connections depends on the slice configuration (capacity and placement of virtual functions) managed by the slice manager, which, in turn, consumes resources (computing and connectivity). Note that the placement of the functions cannot be conducted in any potential location site due to the constraints of each RAN segment, such as distance between sites and latency requirements [26]. Table 2 summarizes the mapping of the virtual functions and site types, based on a typical network operator configuration [22]. In the case of DU and assuming split 7.2 for F-H, only MBS and ACOs are suitable for its deployment. However, M-H latency can be relaxed by means of split 2, which allows the extension of its placement to RCO if suitable, i.e., for eMBB services. Regarding UPFs, without loss of generality, we assume that they consist of processes that require more intensive computation and centralization than those of DU/CU. Therefore, due to the very limited availability of resources at the MBS, the placement of such functions is avoided at the very edge of the network. In addition, although function placement is allowed in ACOs, their computational resources are reserved for URLLC and mIoT services due to their limited capacity.

## 3. B5G RAN and Slice Operation

As introduced in Section 1, smart slice operation is built upon three main pillars: (*i*) *dynamic µBSs management*, by switching the µBSs on and off, with the objective of reducing energy consumption in the RAN, while ensuring the minimum capacity needed to support UE traffic; (*ii*) *dynamic RAN capacity slicing*, with the aim of managing physical radio blocks (PRBs) to assign resources to each of the different slices in order to provide the required QoS; and (*iii*) *flexible functional split operation*, where the placement of virtual functions (DU/CU) is adapted to match the requirements of the UE served by each BS in a cell. In this section, we aim to illustrate how that smart slice operation dramatically affects the traffic supported by the underlying transport network in each of the segments of the reference topology.

**Figure 3:**
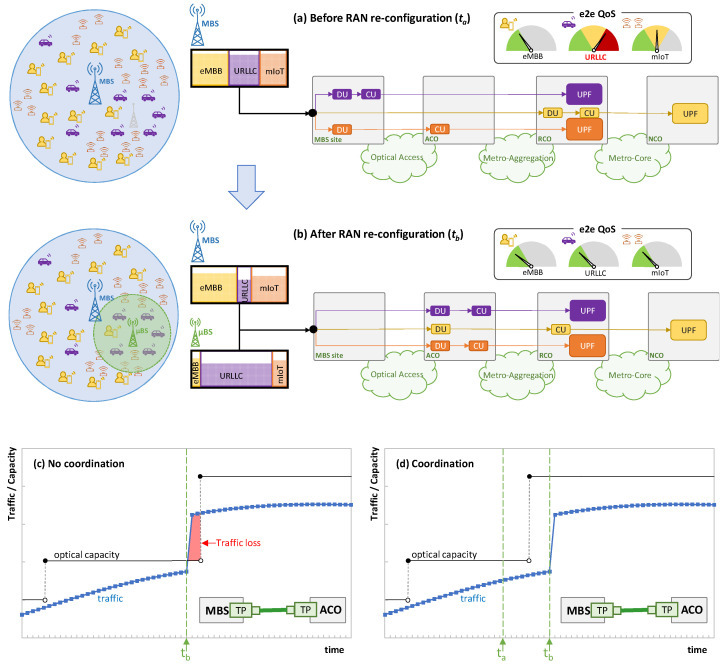
Example of RAN reconfiguration before (**a**) and after (**b**) BS activation and function placement reconfiguration. Capacity allocation in optical access without (**c**) and with (**d**) RAN-fixed network coordination.

Figure 3a shows the RAN state at a given time *t_a_* of an example consisting of one cell with one active MBS that provides connectivity to a mix of UE from different services. For the sake of simplicity, we assume that one slice per service type is deployed. Let us assume that the core network orchestrator decides, at the slice provisioning time, the placement of UPF according to slice type and QoS requirements. This UPF placement remains fixed during the slice’s lifetime. Moreover, each slice has its own placement of DU/CU along the different CO sites (see Figure 1b). In this case, DU/CU placement can be dynamically reconfigured by the slice manager according to current and expected UE traffic conditions in order to guarantee that e2e latency (i.e., from UE to UPF) meets the requirements of the slice service type. The figure also shows a simplified view of the PRBs used by each of the slices. Table 3 shows the RAN segment per service class that is transported in each of the network segments. Note that the traffic in each segment is a heterogeneous mix of F-H, M-H, and B-H traffic, depending on the slice configuration. The selected time instant *t_a_* illustrates a scenario in which the radio resources are reaching a point of saturation that is negatively affecting the e2e service QoS (represented by colored gauges). In particular, the URLLC service is strongly affected by such saturation, even when the DU/CU functions are currently placed as close as possible to the UE to reduce e2e latency. In view of this, let us imagine that such QoS degradation is detected by the slice manager and that, after analysis, some *slice reconfigurations* have been identified; consequently, there are several actions to be performed. First, the slice manager triggers the activation of an available µBS (in light grey in Figure 3a). Due to the physical location of the antenna and its proximity to the majority of the URLLC UE, the activation of this new antenna relieves the MBS from having to serve most of the URLLC traffic. Figure 3b shows the RAN state after the activation of such a µBS at time instant *t_b_*. As the activated µBS (now in green) captures most of the URLLC traffic, the overall RAN load is reduced and, consequently, the delay introduced by the RAN segment [17], which in turn makes the e2e QoS of all the services reach the desired target performance.

Nonetheless, smart slice operation goes beyond µBS activation. For instance, in order to reduce the cost associated with virtual function placement, URLLC and mIoT functions can be now located far from the edge (where the available resources are typically cheaper) without major impact on the QoS of those services. This action might require the reallocation of some of the functions of the other slices, for the sake of global optimization (as illustrated with the reallocation of the eMBB functions). Therefore, as a consequence of this smart slice reconfiguration (of both µBS activation and virtual function reallocation), the traffic supported in the fixed network segments sharply changes. Table 4 updates Table 3 after slice reconfiguration, where we observe segments that are greatly affected, e.g., the traffic in the optical access sharply increases due to the addition of large F-H traffic volumes.

It is worth noting that the change in the transport network traffic between time *t_a_* and *t_b_* cannot be predicted by typical monitoring and data analytics control loops in the fixed network [15], since the reason for that change is uncorrelated with the previously observed traffic. To better illustrate this problem, Figure 3c sketches the autonomous operation of an access optical connection between the MBS site and the ACO following a typical control loop, such as the one proposed in [15], and in the event of the smart RAN operation exemplified in Figure 3a,b. Based on traffic prediction, optical capacity (in the granularity of optical subcarrier units) can be smoothly adapted to traffic changes so that the service is guaranteed and the capacity (and transponder energy consumption) is minimized. However, due to the lack of coordination between network domains, once the RAN reconfiguration is performed (at time *t_b_*), the traffic suddenly increases in the optical access, which can lead to traffic loss until the optical capacity is reactively extended. Hence, in order to overcome this problem, some *contextual information* about the slice operation needs to be provided to the transport network orchestrator before it actually happens (at time *t_a_*) so that the latter can prepare the fixed transport network accordingly. This is illustrated in Figure 3d, where the anticipation of the RAN reconfiguration can be added to the process of predictive optical capacity allocation. Note that, according to this assumption, the optical capacity can be increased before the optical access traffic actually changes. Although this action might produce some overprovisioning of the optical capacity between times *t_a_* and *t_b_*, the benefits in terms of traffic loss mitigation outweigh the need for coordination.

In view of the above, the next section proposes a *context-aware autonomous network operation* solution based on the sharing of contextual information between the slice manager and the fixed transport network orchestrator in order to allow AI-based autonomous operation to efficiently control the optical capacity allocated to the optical connections supporting e2e slice connectivity.

## 4. Context-Aware Autonomous Network Operation

**Figure 4:**
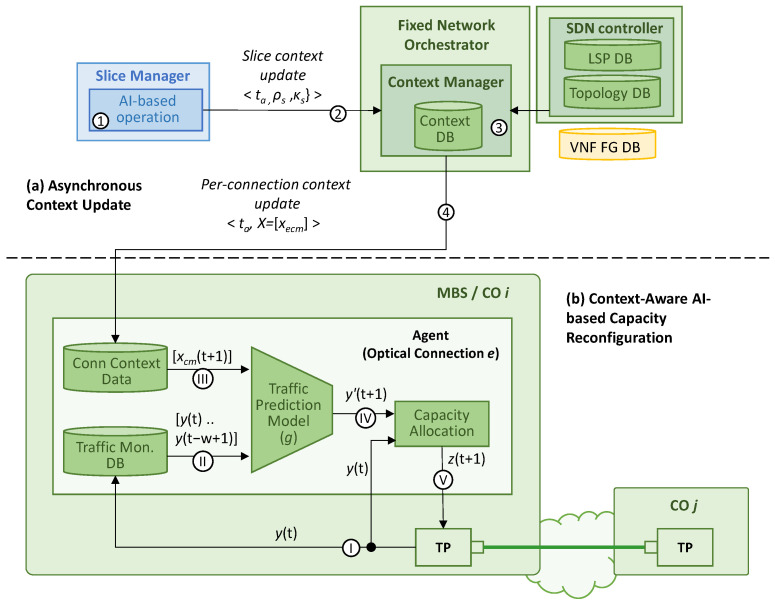
Context-aware autonomous network operation scheme containing: asynchronous context update (**a**) and context-aware AI-based capacity reconfiguration (**b**).

Figure 4 sketches the architecture and workflows of the proposed context-aware autonomous network operation. By means of this procedure, the RIC shares with the fixed network orchestrator the relevant information about the slice reconfigurations that come before the slices changes are actually performed. With this relevant input, the transport network orchestrator can generate a context for the different agents running autonomous network control loops. In particular, those agents are in charge of dynamically allocating capacity to the optical connection according to the current and predicted traffic [15]. Although the procedure is focused on the reconfiguration of existing slices, note that it can be easily extended to the provisioning of new e2e slices, i.e., a first reconfiguration notification can be the provisioning of the slice. Table 5 introduces the notation consistently used in this section.

The proposed procedure can be divided into two main processes. Figure 4a illustrates the *asynchronous context update workflow* that is executed when the slice manager decides to perform a reconfiguration, which is assumed to happen at time *t_a_* (labeled as 1 in Figure 4a). Let us assume that that reconfiguration decision is computed by an AI-based operation module (e.g., one that is similar to that of [2]) that analyzes the slice monitoring data and predicts a new (better) configuration for one or several e2e slices. Without loss of generality, we assume that such a reconfiguration is performed at a given time *t_b_* > *t_a_*. For instance, the AI-based module can predict an increase in UE activity and decide, at time *t_a_*, that a new µBS in a given cell is needed 5 min later. Consequently, the capacity of some slices increases accordingly once the µBS is active, i.e., at time *t_b_* = *t_a_* + 5 min. Note that, in the case of a reactive RAN reconfiguration due to some unexpected and unpredictable event, it might happen that *t_b_* = *t_a_* limits the performance of the context-aware operation in the short period of time between the performance of the reconfiguration and the convenient updating of the context.

As soon as the decision is taken, a *slice reconfiguration notification* is sent to the context manager at the fixed network orchestrator for each of the slices that are to suffer some modification (2). This notification for slice *s* contains the time where the reconfiguration is expected to happen (*t_b_*) and the new characteristics of the slice, namely the capacity *κ_s_* (in capacity units like the number of PRBs) allocated to the radio segment and the graph *ρ_s_* with the placement of each virtual function *f* ∈ *F*(*s*) in the set of computing nodes *V*. Although both characteristics are related to RAN configuration, they are also indicative of the actual UE activity and, consequently, correlated with the actual slice traffic. Hence, this notification procedure enables the passing of relevant information from the RAN to the fixed network in an asynchronous and efficient way, without the need of continuous monitoring data exchange between domains.

Once the context manager receives a slice reconfiguration notification, it executes the context computation process (3). This process aims at computing the changes in the context due to the new reconfiguration. In a timely manner, those changes are updated in each of the agents managing optical connections (4), in order to guarantee that they can use up-to-date contextual information when running autonomous operation actions. Recall that the slice manager triggers the needed reconfiguration workflows with sufficient anticipation to ensure that they are active at time *t_b_.*

More formally, we denote *X* as the context variable set at a given reconfiguration time *t_b_*. This variable contains, for each optical connection *e* ∈ *E*, the expected capacity (in normalized capacity units) that such a connection will support for each service class *c* ∈ *C* and segment *m* ∈ *M.* More formally, each *x_ecm_* ∈ *X* can be expressed as follows:

(1) $$x_{ecm}=\underset{s\in S}{\sum }\delta_{sc}\underset{l\in L\left(s\right)}{\sum }\delta_{lm}\cdot \delta_{le}\cdot \kappa_{s}$$

Note that this context omits particular details of the individual slices since it aggregates all the slice capacity per class and segment. Thus, the privacy of individual slices (clients) is guaranteed when operating optical connections. Moreover, in order to compute the context variables, static information about the mapping of slices to classes (*δ_sc_*) and links to segments (*δ_lm_*) is needed from the slice definition, which can be easily obtained at slice provisioning time. However, the mapping of slice links to optical connections (*δ_le_*) is dynamic and depends not only on the slice graph *ρ_s_* but also on the placement of the computing nodes in the COs (*δ_vn_*), which can change in time depending on the fixed network reconfiguration actions.

Algorithm 1 details the context computation process, which is required for every single slice reconfiguration notification. Thus, the algorithm receives all the data of one slice reconfiguration notification at a given time *t_b_*, computes the new context for that time, and updates the context of those already planned reconfigurations that will happen at time *t_b_* (and after it). Moreover, the process has access to the internal DB that stores the static slice characteristics, context status, and transport network configuration and is conveniently kept up to date by the fixed network controller.

Note that the DB can store multiple context variable sets and fixed network configurations if several reconfigurations are planned to happen at different times. Then, for convenience, the slice reconfiguration and context data are stored and indexed by time; so, they can be easily retrieved and modified every time a new reconfiguration notification is processed. For those slice static parameters, we assume that they follow a non-temporal indexing by slice id.
**Algorithm 1:** Context computation (Context Manager)**INPUT**: *s*, *t_b_*, *ρ_s_*, *κ_s_* **OUTPUT:** -1: 2: 3: 4: 5: 6: 7: 8: 9: 10: 11: 12: 13: 14: 15: 16: 17: 18: 19: 20: 21:**if** *DB*[*t_b_*] == ∅ **then**
  *t*′ ← getPreviousTime(*DB*, *t_b_*)
  *DB*[*t_b_*] ← *DB*[*t*′]
*t*″ ← getHighestTime(*DB*)
*T* ← getTimeRange(*t_b_*, *t*″)
*c* ← *DB*[*s*][“c”]
**for each** t_i_ ∈ T **do**
  *X*={*x_ecm_*} ← *DB*[*t_i_*][“*X*”]
  *κ*^0^ ← *DB*[*t_i_*][“*κ_s_*”]
  *π_s_*^0^ ← *DB*[*t_i_*][“*π_s_*”]
  *π_s_* ← computeMapping(*ρ_s_*) (Algorithm 2)
  **for each <***l*, *e*> ∈ *π_s_*^0^
**do**
     *m* ← *DB*[*s*][*l*][“m”]
     *x_ecm_* ← *x_ecm_* − *κ*^0^
  **for each <***l*, *e*> ∈ *π_s_* **do**
     *m* ← *DB*[*s*][*l*][“m”]
     *x_ecm_* ← *x_ecm_* + *κ_s_*
  *DB*[*t_i_*][“π_s_”] ← *π_s_*
  *DB*[*t_i_*][“κ_s_”] ← *κ_s_*
  *DB*[*t_i_*][“*X*”] ← *X*
**return -**

The algorithm starts by generating a new entry in the DB for time *t_b_* (if it does not exist yet), copying the same data in the entry indexed in the immediately previous time to *t_b_* (lines 1–3 in Algorithm 1). Then, the vector of times *T* is obtained; this vector contains the list of available times in the DB between *t_b_* and the highest available time (lines 4–5). In other words, this identifies all the different contexts that need to be updated due to the new reconfiguration. Next, after retrieving the class of the slice, a loop is executed to update all the contexts (lines 6–7). Thus, for each *t_i_* ∈ *T,* the stored context *X*, capacity *κ*^0^, and mapping *π_s_*^0^ of the slice links to the optical connections are retrieved from the DB (lines 8–10). Then, the new mapping *π_s_* is computed from graph *ρ_s_* (line 11). After this computation, the stored slice capacity is subtracted from all the *x_ecm_* variables belonging to the stored mapping (lines 12–14), whereas the new capacity is added to the *x_ecm_* variables of the new mapping (lines 15–17). Finally, the slice capacity, mapping, and context are updated (lines 18–20).

Algorithm 2 details the process of computing a mapping *π_s_* from a new slice graph *ρ_s_*, i.e., line 11 of Algorithm 1. After some initialization, the process iterates in the graph in order to obtain the pair of tuples <*f*,*v*> and <*f’*,*v’*> that identify each graph adjacency (lines 1–4 of Algorithm 2). Then, queries to the DB are performed to retrieve the slice link *l* that supports that graph adjacency (line 5), as well as the optical connection *e* that connects the sites where the computing nodes are running (lines 6–8). Afterwards, the tuple <*l*,*e*> is added to *π_s,_* which is eventually returned (lines 9–10).
**Algorithm 2** Compute Mapping**INPUT**: *ρ_s_* **OUTPUT**: *Π*1: 2: 3: 4: 5: 6: 7: 8: 9: 10:*π_s_ =* []
**for**
*i =*1..*|ρ_s_|* − 1 **do**
 *<f*, *v>* ← *ρ_s_*[*i*]
 *<f’*, *v′>* ← *ρ_s_*[*i + 1*]
 *l* ← *DB*[*s*].query(“*l*” | *δ_lf_* == 1 & *δ_lf’_* == 1)
 *n* ← *DB*[*s*].query(“*n*” | *δ_vn_* == 1)
 *n′* ← *DB*[*s*].query(“*n*” | *δ_v_*_′_*_n_* == 1)
 *e* ← *DB*.query(“*e*” | *δ_ne_* == 1 & *δ_n__′__e_* == 1)
 *π_s_.*add(<*l*,*e*>)
**return**
*Π*

As already mentioned, in addition to the asynchronous context update workflow, the *context-aware AI-based capacity reconfiguration* workflow (Figure 4b) is executed periodically at every time *t*, i.e., when a new traffic monitoring sample *y*(*t*) is collected (labeled as I in Figure 4b). To support this workflow, the following elements are deployed in the agent: (*a*) the traffic monitoring DB storing the last *w* traffic values; (*b*) the context DB with the current and future contexts affecting the optical connection; (*c*) the traffic prediction model that estimates the expected traffic *y*(*t* + 1); and (*d*) the capacity allocation module that decides the amount of optical capacity units *z*(*t* + 1) (e.g., digital sub-carriers) to be configured in the optical transponder according to traffic prediction *y*(*t* + 1) to stay below a desired (target) maximum load.

Once the new measurement is collected, the traffic monitoring DB is updated (II) and the expected context at the prediction time, i.e., *t* + 1, is retrieved from the context DB (III). For convenience, since the connection context DB contains only context variables for the specific connection, we removed the sub-index *e* from the *X* variables. Then, the traffic prediction model *g* can be formally expressed as follows:

(2) $$y’\left(t+1\right)=g\left(\left[y\left(t-i\right),\forall i=0..w-1\right],\left[x_{cm}\left(t+1\right),\forall c\in C,m\in M\;\right]\right)$$

which results in *w + |C|* × *|M|* inputs and provides one single output (IV). The training model *g* can be autonomously conducted by the agent as soon as it receives context variables and can learn from them. Without loss of generality, we assume that the agent, at its provisioning time, receives an initial model that has been trained offline with generic traffic data and null context inputs. Then, the model is re-trained online, combining the contextual variables and monitored traffic and improving the accuracy of the initial one.

The traffic prediction *y′*(*t* + 1) is then processed by the capacity allocation module (V), jointly with the current traffic *y*(*t*). Thus, with *λ_max_* as the target maximum load, *u* as the traffic supported by each optical capacity unit, and *z_max_* as the maximum number of optical capacity units supported by a transponder, *z*(*t* + 1) can be formally defined as follows:

(3) $$z\left(t+1\right)=\max\left(z_{max},\frac{\max\left(y\left(t\right),y’\left(t+1\right)\right)}{\lambda_{max}\cdot u}\right)$$

Without loss of generality, we assume that the transponder internally implements an agent that allocates the target number of capacity units detailed in *z*(*t* + 1) [15]. Note that, while model *g* is being retrained online and the *y′*(*t* + 1) predictions have not reached a target accuracy performance, a more conservative policy can be temporarily used for capacity allocation, e.g., *z*(*t* + 1) = *z_max_*.

As a final remark, it is worth mentioning that the proposed context-aware approach does not increase execution time with respect to the existing optical capacity management approaches based on optical connection monitoring and local decision making [15]. Note that the connection context is stored and available at the local agent and is simply added as another input to the prediction model. Moreover, this connection context is updated asynchronously, e.g., every time a slice reconfiguration update is processed by the context manager. Thus, context management and AI-based optical capacity reconfiguration are completely independent processes; therefore, the former does not increase the computing time of the latter.

## 5. Illustrative Results

In this section, we first describe the details of the simulation environment and network configuration used for the performance evaluation purposes. Then, the smart slice operation implemented in the simulator is described in detail. Afterwards, the context-aware autonomous network operation in Section 4 is evaluated by means of two numerical studies. On the one hand, the performance of the connection traffic prediction using contextual information is analyzed and compared against a benchmarking approach. On the other hand, the proposed capacity reconfiguration method is validated for different operational objectives.

### 5.1. Simulation Setup

A Python-based simulator was implemented based on the one presented in [17], where a flow-based network simulator was proposed as an accurate and efficient tool for reproducing network scenarios that mix RAN and fixed network technologies. In a nutshell, the simulator generates flow traffic by means of statistics-based generators that emulate RAN cells and fixed access points and propagate them through a system of fluid-flow continuous queues that model the different network elements (packet and optical interfaces and connections) and segments (RAN cell, optical access, etc.).

**Figure 5:**
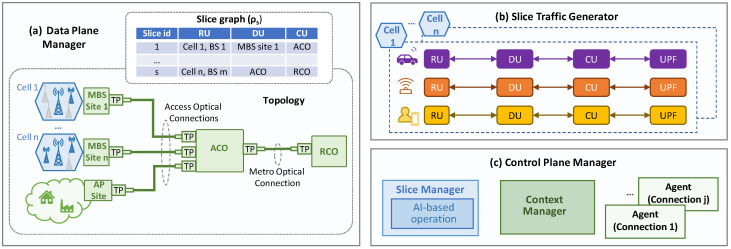
Simulator blocks and components.

Figure 5 shows the main blocks and components implemented in the simulator. It contains a *data plane manager* (Figure 5a) that simulates a topology consisting of a number of RAN cells, all of them individually connected to a reference ACO by means of *access optical connections* (i.e., each optical connection transports traffic from a single RAN cell). Then, the traffic received at the ACO (that aggregates several RAN cells and the fixed access traffic) is propagated to the reference RCO by means of a *metro optical connection*. Without loss of generality, we assumed point-to-point optical connections, each supported by a number of sub-carriers of 25 Gb/s each [15]. In conjunction with this topology, a set of slices is served, each identified by the graph *ρ_s_*, which allows the mapping of each slice function with a computing node (we assume one single computing node per MBS and CO).

The available RAN traffic models in [17] are based on 4G technology and do not consider either functional splitting or slicing, i.e., the traffic injected by the RAN cells is always B-H. As these models are not valid for the considered smart RAN operation, we developed a novel RAN *slice traffic generator* (Figure 5b) that, given a specific RAN cell configuration, generates synthetic slice traffic flows for each of the considered service classes (eMBB, URRLC, and mIoT) and each slice link (radio, F-H, M-H, and B-H). Note that this traffic can be easily mapped to each of the network segments according to the placement of the DU and CU functions maintained in the data plane manager. The source code of the generator is openly available in [27], where instructions to generate the main data used in this section are provided, as well as indications about how to reproduce other configurations and policies. Finally, the *control plane manager* (Figure 5c) implements the different modules depicted in Figure 4 that are related to context-aware operation, namely: (*i*) the slice manager containing the AI-based operation module; (*ii*) the context manager and related DBs at the fixed network orchestrator; and (*iii*) a connection agent for each access and metro optical connection.

**Figure 6:**
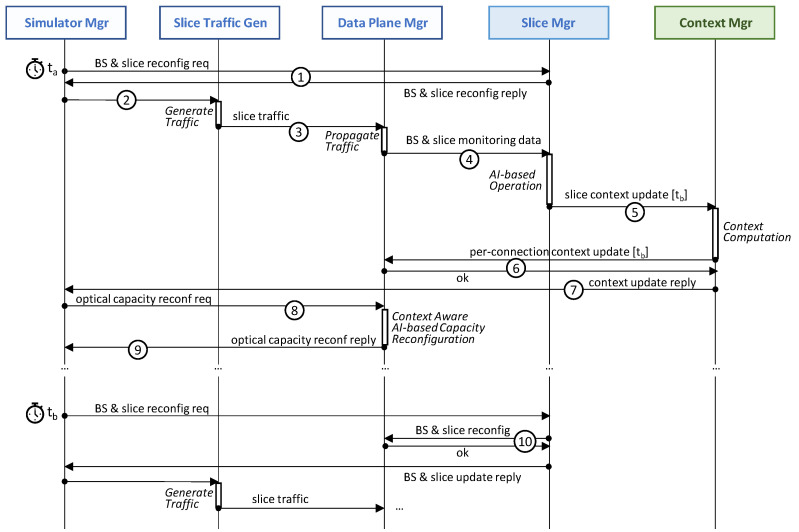
Simulator workflow.

Figure 6 shows the simulator workflow that is periodically executed after every minute of simulated time; it shows the blocks and components in Figure 5 as well as the simulator manager that controls the execution of the workflow at every time step. At a given time step *t_a_*, the simulator manager first asks the slice manager to perform the programmed RAN slice reconfiguration decided in a previous time step, if one exists (labelled as 1 in Figure 6). In the depicted case, no changes need to be applied, and the reply is sent back to the simulator manager. Then, the simulator manager triggers slice traffic generation for the current time step *t_a_* (2), which is propagated through the topology that is assumed in the current graph *ρ_s_* (3). The result of this propagation generates relevant data for performance evaluation purposes (which, for simplicity, are not depicted), as well monitoring data that are collected by the slice manager in charge of the AI-based operation actions (4). These actions, which are explained in detail in Section 5.2 below, can generate slice context updates that need to be announced to the context manager in a timely manner (5). Upon the reception of a context update, the context manager computes per-connection context updates that are distributed to the agents controlling the optical connections in the data plane (6). After receiving confirmation from all the agents, a context update reply is sent back to the simulator manager (7).

The last phase to reproduce at every time step involves the reconfiguration of the optical capacity of the connections with the prediction expected for next time step *t* + 1, which now can be performed with an updated context (8). Thus, the agents of the connections perform context-aware AI-based capacity reconfiguration, as explained in Section 4, and then notify the manager when finished (9). This concludes the execution of a time step. Note that, at time step *t_b_*, the request of the BS and slice reconfiguration produces changes in the network, which need to be executed (10) before continuing with the traffic generation and propagation phases.

### 5.2. RAN Configuration and AI-Based Operation

**Figure 7:**
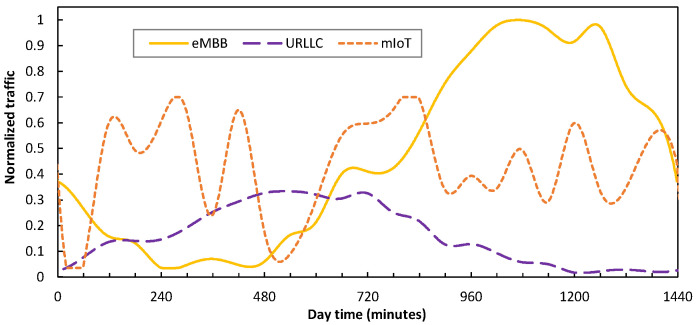
UE traffic per service class.

Regarding RAN configuration, we considered RAN cells consisting of 1 MBS working at the sub-6 GHz band (2 × 2 MIMO, 20 MHz bandwidth, 30 KHz subcarrier spacing, and 55 PRBs), and a variable number of µBSs configured in the mmWave band (8 × 8 MIMO, 100 MHz bandwidth, 30 KHz subcarrier spacing, and 275 PRBs). Without loss of generality, we assumed that every active BS has one slice per service class. Moreover, the F-H and B-H traffic were computed according to the formulation and references in [18]. Figure 7 illustrates the UE traffic demand per service class (normalized to maximum) in a typical day as a function of time; this was obtained according to the assumptions in [22] and [28] for a medium-term scenario. As can be seen, eMBB presents a clear peak in the afternoon/evening time, whereas URLLC inversely increases in the morning/noon period. Regarding mIoT, it fluctuates with peaks and valleys distributed throughout the whole day. Overall, the percentages of the traffic of each type are 45%, 15%, and 40% for eMBB, URLLC, and mIoT, respectively.

Two different RAN cell scenarios were configured according to the distribution of UE within the cell areas. For the sake of a fair comparative analysis, the total demand and the demand per service class are the same in both scenarios. Then, in the *clustered* scenario, the UEs types of the same class are grouped in a given region within the area and separated from the other classes. This means that a given µBSs will typically provide service to (mainly) the UE of one single class. Therefore, the clustered scenario tends towards µBSs with a predominant slice and a more variable load (depending on the predominant class) during the day. On the other hand, the *distributed* scenario assumes that the UEs types are randomly placed in the cell area and, therefore, that there are no clusters of UE of the same class. Consequently, all the µBSs have a similar load (which fluctuates with the overall traffic) with a mix of slices from all the service classes.

As described in Section 3, the main objective of the smart slice operation is to minimize capacity utilization by adjusting the number of active µBSs and the slice capacity (which leads to energy savings) while ensuring the minimum capacity needed to support UE traffic and guarantee a committed QoS of the different slices. Thus, we implemented a policy to run in the slice manager that consisted of rules oriented towards reconfiguring the RAN capacity resources and slices by means of the computation of traffic loads (defined as UE traffic over RAN capacity), and we compared them with thresholds in order to determine the actions to perform. Whenever an action is detected at time *t_a_*, slice reconfiguration notification (if needed) is created with time *t_b_* = *t_a_* + 1, thus giving time for a context update and the proper use of contextual information for AI-based optical connection operation. Note that the slice reconfiguration actions detected and notified at time *t_a_* are actually performed at time *t_b_*.

We conducted an exhaustive set of simulations in order to find the configuration of rules and thresholds that produces a remarkable energy consumption reduction (measured in terms of active µBS) that guarantees robust QoS (no traffic loss). Specifically, the rules and actions that have been configured for each of the three pillars identified in Section 3 are the following:

- *Dynamic µBSs management*: when one µBS exceeds a total load of 75%, the closest inactive µBS (if any) is switched on. Otherwise, if the load drops below 15% and the neighbor µBSs are active, then the µBS is switched off and its current demand is then served by its active neighbor µBS.
- *Dynamic slice capacity*: the PRBs assigned to each slice are assigned and released in order to keep (or approach) a slice load between 60% and 70%.
- *Flexible functional split operation*: depending on the number and utilization of the µBSs, we define both *low-load* and *high-load* regimes, in which different splits and virtual function placements are considered. Table 6 details the different regimes for each service class. For instance, the URLLC slices follow a semi-centralized approach (DU at ACO and CU at RCO) when the RAN cell load is below 30%. When the load exceeds that threshold, the functions approach to the UEs to reduce the risk of a potential delay degradation due to high load (DU at MBS and CU at ACO).

**Figure 8:**
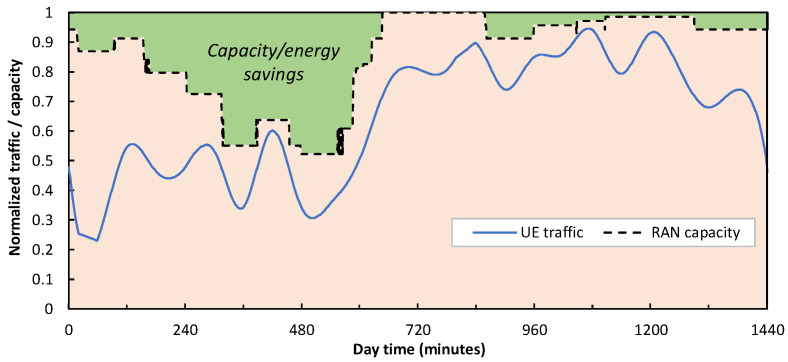
UE Smart RAN capacity allocation.

**Figure 9:**
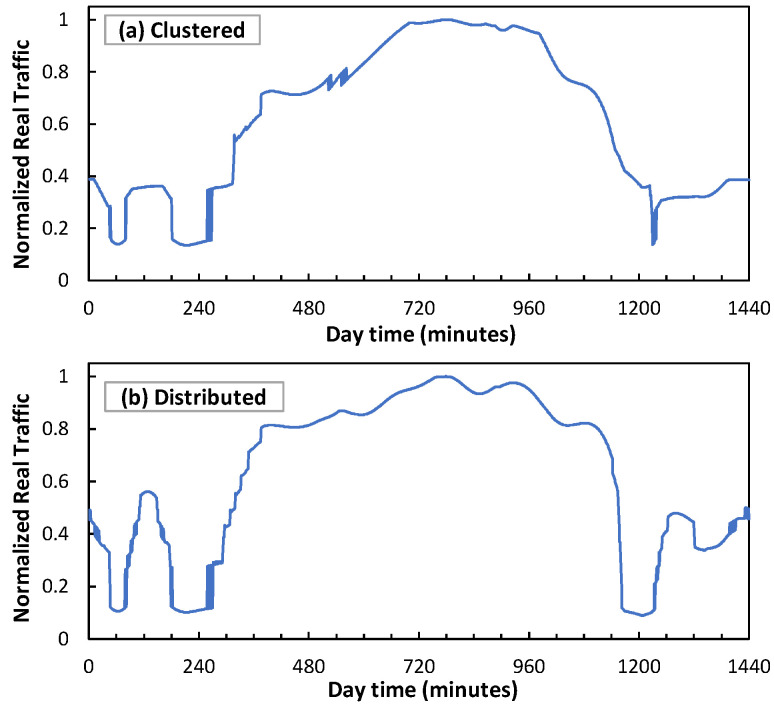
Access optical connection traffic for clustered (**a**) and distributed (**b**) scenarios.

Figure 8 shows the total allocated RAN capacity (as a result of the number of active µBSs and the PRBs allocated to each slice) of a cell with 1 MBS and 16 µBSs serving the mix of UE traffic in Figure 7. Note that no traffic loss is experienced by the UE (the total traffic is always below capacity), which validates the proposed RAN operation. Moreover, the RAN capacity resources are smoothly adapted to demand, which results in energy savings [18]. To demonstrate a direct consequence of such a smart slice operation, Figure 9 shows an example of traffic injected into an access connection for both clustered and distributed scenarios. As can be seen, sudden changes are observed which are much sharper than that of the UE traffic in Figure 7.

Hereafter, we assume that the RAN operates according to this policy and proceed to evaluate the context-aware autonomous network operation in Section 4.

### 5.3. Optical Connection Traffic Prediction

As previously stated, this section is devoted to showing the accuracy of the optical connection traffic prediction model that uses RAN slice context data; this model is compared with a benchmarking approach where the optical network autonomous operation is performed in the absence of RAN-fixed network coordination. This study is conducted for both access and metro optical connections and for both distributed and clustered scenarios.

Let us start by focusing on the traffic prediction carried out in the agent of an access optical connection connecting the MBS of a RAN cell with 1 MBS and 16 µBSs with its associated ACO site. In particular, we aim to evaluate the accuracy of predicting *y*(*t* + 1) using the model in Equation (2) (named as *context*) and to compare it with the model used in [20] (named as the *benchmark*) that showed high accuracy in predicting traffic in fixed networks supporting 4G and residential services. Note that the benchmark model predicts *y*(*t* + 1) only as a function of the last *w* traffic values stored in the monitoring DB. Both models have been implemented as deep neural networks with 2 hidden layers with 24 and 12 neurons, respectively, and hyperbolic tangent activation functions. Thus, the comparison between both models enables a fair way to evaluate the use of contextual data in B5G scenarios.

**Figure 10:**
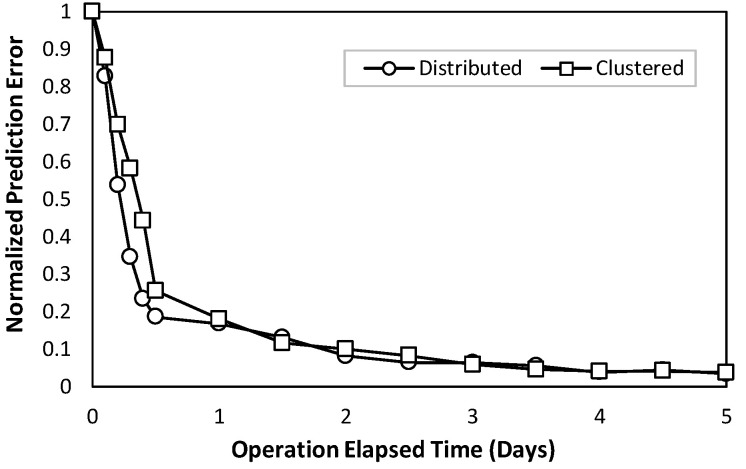
Online training performance of context traffic prediction model.

Our first numerical evaluation concentrates on the training of the context-based model at the provisioning time of a given optical connection, i.e., when the connectivity between a RAN cell and the ACO is established for the first time. At this provisioning time, as already mentioned in Section 4, an initial model trained with generic traffic data and null contextual variables is loaded into the connection agent. Then, as soon as the real traffic measurements and connection context variables are received, the prediction model is retrained online until the prediction error reaches a stable value. Figure 10 shows the normalized prediction error (normalized to zero-day error) as a function of the elapsed operation time (in days) from the provisioning time instant. As can be seen, for both the distributed and clustered scenarios, convergence to a stable error (one order of magnitude lower than that of the initial model) is reached after few days of operation (3 to 5). As the connectivity between RAN cells and ACOs typically lasts for very long time periods (weeks to months), the duration of this initial tuning phase is negligible with respect to optical connectivity lifetime. Thus, context-based prediction models can be practically deployed since they are easily trained and remarkably improved with the data available in the optical connection agent.

**Figure 11:**
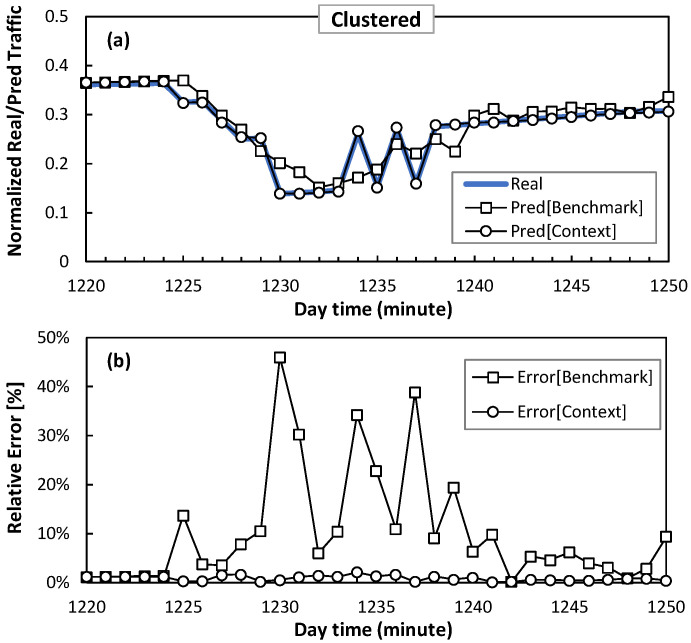
Traffic prediction detail in access optical connection for clustered scenario in terms of absolute (**a**) and relative (**b**) error.

**Figure 12:**
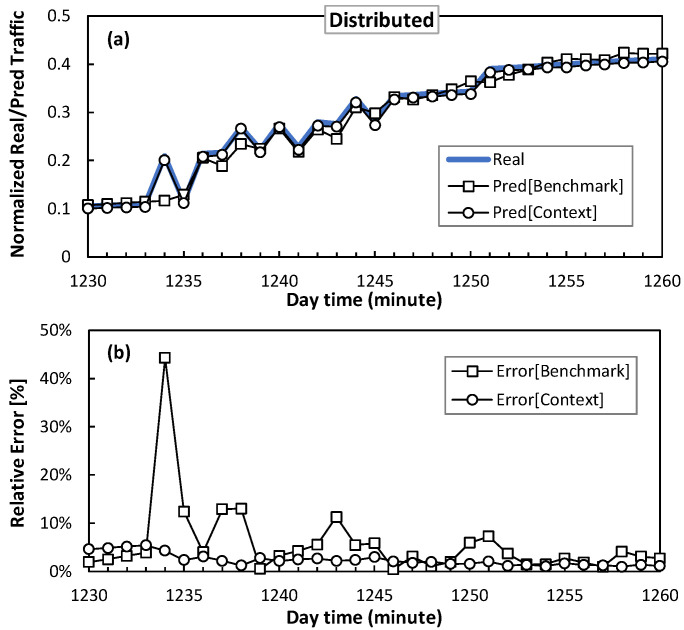
Traffic prediction detail in access optical connection for distributed scenario, in terms of absolute (**a**) and relative (**b**) error.

Next, regarding the error convergence performance obtained in Figure 10, where a stable prediction error for both the benchmark and context models is reached, Figure 11 and Figure 12 show a detail of the performance of the traffic prediction for one of the sudden traffic changes introduced by smart slice operation in clustered and distributed scenarios, respectively. The figures show real traffic and predicted traffic using both benchmark and context methods, as well as the relative error. Note that benchmark prediction presents large inaccuracies (errors around 50%) in the presence of those sudden changes. Conversely, the context model provides accurate and smooth prediction since it anticipates sudden changes thanks to context information.

Table 7 summarizes the average and maximum error during the whole day, for both models, using mean square error (MSE) and relative error as accuracy metrics. Moreover, the reduction in error (in %) that the context model provides with respect to the benchmark one is detailed. In view of these results, we can conclude that the context model clearly improves the benchmarking one by remarkably reducing both average and maximum error. Especially interesting is the case of the maximum relative error: the context model reduced 93% and 89% of the error of the benchmark model for the clustered and distributed scenarios, respectively.

**Figure 13:**
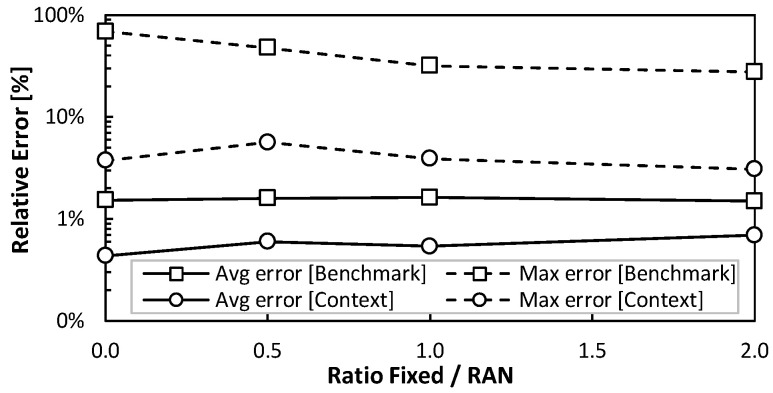
Traffic prediction in metro optical connection.

**Figure 14:**
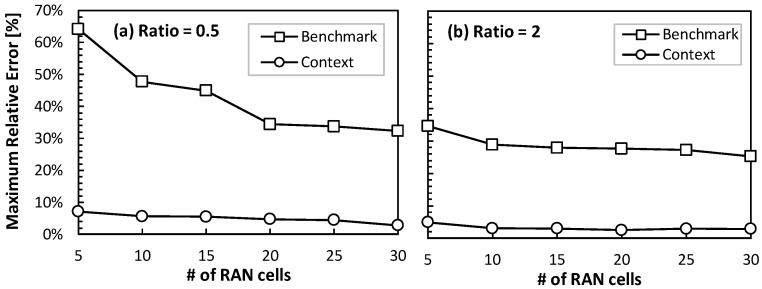
Maximum error for ratio 0.5 (**a**) and 2 (**b**).

Let us now focus on evaluating the accuracy of the traffic prediction for a metro optical connection connecting an ACO with its reference RCO. Recall that this type of connection supports traffic from a number of RAN cells, as well as traffic from fixed AP sites (which typically show smoother daily patterns when mixing residential and business traffic). Figure 13 shows the average and maximum error of a metro optical connection that supports 10 RAN cells (mixing clustered and distributed scenarios) and a variable number of fixed APs. In particular, we defined several configurations with different ratios of fixed/RAN (1 means that the fixed and RAN traffic volumes are equal; 2 means that the fixed traffic is double than that of the RAN). It is worth noting that the improved prediction by the context model persists even for large ratios (i.e., when the overall traffic becomes more predictable). In other words, the sharp fluctuations induced by the RAN operation have a large impact on the aggregated flows, which validates the extension of the context update to the metro optical connections (and not only access ones). Figure 14 depicts the evolution of the maximum relative error as a function of the number of RAN cells for ratios 0.5 and 2. In line with the previous results, the error reduction achieved by the context model is outstanding. Indeed, the benchmark model produces relative maximum errors of 30% in optical connections supporting large traffic volumes, which can have a large impact in terms of absolute errors (dozens to hundreds of Gb/s).

In view of these results, we can conclude that using the RAN slice context significantly increases the accuracy of the models predicting the traffic supported by optical connections. In terms of the reduction in maximum error, the context model reduces the error of the benchmarking model by between 85% and 93% when predicting traffic from access connections. Regarding metro connections, where the RAN and fixed access traffic is mixed, the benchmarking model provides a much larger prediction error (~30%) than the context one (~2%), even when the fixed access traffic doubles that of the RAN traffic.

### 5.4. Optical Connection Capacity Reconfiguration

Now that the accuracy of the context model has been validated and its improvement with respect to benchmark one has been shown in terms of the prediction error, let us finally focus on evaluating the impact of such predictions to dynamically allocate optical connection capacity. For the sake of simplicity, we focus on access connections and evaluate the size of the RAN cells for the distributed scenario, i.e., with the number of μBSs ranging between 16 and 128, to consider the different cell densities [29]. Moreover, we consider two different operational objectives to be configured in the capacity allocation module in the connection agent. On the one hand, the *capacity minimization* objective aims at adjusting the allocated optical capacity so that it is as close as possible to the actual traffic. Thus, the load of optical connections can grow up to 95% in order to exploit the optical capacity resources. On the other hand, the *delay reduction* objective allocates an excess of capacity to avoid congestion in the fixed segment, which could introduce additional delay to the RAN slices. Therefore, the maximum load is kept below 80%.

**Figure 15:**
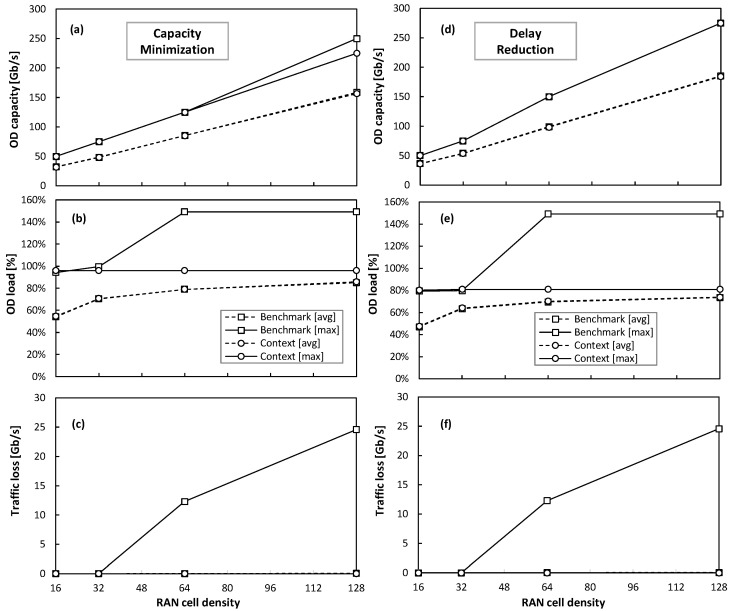
Performance of optical connection capacity reconfiguration for capacity minimization (**a**–**c**) and delay reduction (**d**–**f**) objectives.

Figure 15a–c and Figure 15d–f show the obtained results for the capacity minimization and delay reduction operational objectives, respectively. The results include the average and maximum values of the capacity allocated, the load, and the traffic loss as functions of the RAN cell density. One initial observation is that both models allocate similar overall optical capacity (with small differences in the averages that are hard to observe). Moreover, the (large) relative errors in model prediction shown in the previous section for the low-density RAN cells (16 μBS) do not produce an effect on the decisions made by the connection capacity allocation module. This is because the absolute fluctuations stay between the size of the optical capacity units (recall that we consider 25 Gb/s sub-carriers); consequently, the benchmark model is still a valid traffic predictor.

However, as soon as the density of the cells increases (which is the expected trend [29]), the real magnitude of the prediction inaccuracies exceeds the sub-carrier capacity. Thus, the benchmark model (sometimes) underestimates the required capacity to support the target load, which leads to unacceptable performance (traffic loss) for the RAN cells that are denser than 32 μBS. Conversely, the optical connection capacity allocation based on the context model prediction provides accurate operation, which is always below the desired target load and eliminates traffic loss, for a wide range of RAN cell densities. In light of these results, we can finally validate the proposed context-aware autonomous network operation procedure, which guarantees smooth and smart fixed transport network operation in the presence and during the activity of the smart B5G slice operation.

## 6. Conclusions

In this work, we focused on the impact of B5G slice operation on the underlying transport network infrastructure in charge of providing computing and connectivity resources. In particular, we analyzed how smart slice operation based on pillars, such as dynamic capacity reconfiguration, adaptive µBS management, and flexible functional split configuration, injects highly variable and hard-to-predict traffic into access and metro fixed networks. Thus, effective autonomous fixed network operation cannot be achieved without coordination between the slice manager and the fixed networks. In order to maximize the autonomous fixed network performance while hiding the sensitive information of the individual slices, we explored the concept of context sharing, which allows the slice manager to asynchronously provide information about the slice reconfigurations to be performed in an aggregated and private way with enough anticipation to synchronize with an autonomous fixed network operation. This slice context is then incorporated into the AI-based prediction models that are used for dynamic capacity allocation in optical access and metro networks.

The proposed architecture and algorithms for context update and usage for prediction purposes in optical connections were validated by means of exhaustive simulations that considered a realistic network operator infrastructure. The results showed that adding the slice context to traffic prediction greatly enhanced prediction accuracy. In particular, the maximum prediction error was remarkably reduced compared with the benchmarking approaches for traffic prediction. This prediction accuracy gain is observed under different RAN demand scenarios with heterogeneous services, as well as for optical access networks carrying only RAN traffic and for metro networks transporting a mix of RAN and fixed (residential and business) traffic.

The value of this prediction accuracy improvement was eventually evaluated for the dynamic allocation of capacity to the optical connections supporting slice links, under two different network operational objectives: capacity minimization and delay reduction. In both cases, the benefits of the proposed context-based operation increased with the size and density of the RAN cells. Therefore, we can conclude that, with the increase in RAN traffic and the density of RAN cells that is foreseen for B5G scenarios, there is a need for context-based coordination to achieve actual e2e smart operation.

## Figures and Tables

**Table 1:** Literature review and relevant contributions.

Reference	Smart RAN Operation	Autonomous Optical Network Operation	5G/B5G RAN + Fixed Networks	Coordination RAN + Fixed Network	Context/Knowledge Sharing
[4,5,7,9]	X				
[11,12,13]			X		
[10]	X		X		
[15]		X			
[19]		X		X	
[20]		X			X
[21]	X				X
Our work	X	X	X	X	X

**Table 2:** Virtualized function placement constraints.

Function	MBS	ACO	RCO	NCO
DU	Yes	Yes	Yes (eMBB)	No
CU	Yes	Yes	Yes	No
UPF	No	Yes (URLLC, mIoT)	Yes	Yes

**Table 3:** Network traffic before reconfiguration (time *t_a_*).

Service Class	MBS <-> ACO [Optical Access]	ACO <-> RCO [Metro Aggregation]	RCO <-> NCO [Metro Core]
URLLC	B-H	B-H	-
eMBB	F-H (7.2)	F-H (7.2)	B-H
mIoT	M-H (2/4)	B-H	-

**Table 4:** Network traffic after reconfiguration (time *t_b_*). Changes w.r.t Table 3 are in boldface.

Service Class	MBS <-> ACO [Optical Access]	ACO <-> RCO [Metro Aggregation]	RCO <-> NCO [Metro Core]
URLLC	**F-H (7.2)**	B-H	-
eMBB	F-H (7.2)	**M-H (2/4)**	B-H
mIoT	**F-H (7.2)**	B-H	-

**Table 5:** Notation.

*N*	Set of MBS sites and COs
*E*	Set of optical connections
*V*	Set of computing nodes
*S*	Set of e2e slices
*F*(*s*)	Set of virtual functions, i.e., DU, CU, UPF, of slice *s*
*L*(*s*)	Set of slice links of slice *s*
*C*	Set of service classes, i.e., eMBB, URLLC, mIoT
*M*	Set of RAN segments, i.e., radio, F-H, M-H, B-H
*δ_ne_*	1, if MBS site/CO *n* is adjacent with optical connection *e*
*δ_vn_*	1, if computing node *v* is located in MBS site/CO *n*
*δ_sc_*	1, if slice *s* belongs to service class *c*
*δ_lm_*	1, if slice link *l* belongs to segment *m*
*δ_lf_*	1, if slice link *l* is adjacent to function *f*
*δ_le_*	1, if slice link *l* is supported by optical connection *e*
*κ_s_*	Capacity (in normalized PRBs) assigned to slice *s*
*ρ_s_* = [<*f*,*v*>]	Node-based graph of slice *s*, consisting of an ordered vector of tuples. Each tuple contains the function *f* and the computing node *v* where the function is placed.
*π_s_* = [<*l*,*e*>]	Link-based graph of slice *s*, consisting of an ordered vector of tuples. Each tuple contains the slice link *l* and connection *e* that supports the link.
*y_e_*(*t*)	Traffic monitored in optical connection *e* at time *t*
*x_ecm_*(*t +* 1)	Capacity (in normalized PRBs) supported by optical connection *e* and belonging to segment *m* of class *c* expected at time *t* + 1
*z_e_*(*t* + 1)	Capacity (in optical capacity units) to be allocated to connection *e* at time *t* + 1

**Table 6:** Flexible functional split configuration.

		eMBB	URLLC	mIoT
Load threshold (low -> high)		60%	30%	50%
Low-Load Regime	Split F-H/M-H	7.2	7.2/2	7.2
	Placement DU/CU	RCO/RCO	ACO/RCO	RCO/RCO
High-Load Regime	Split F-H/M-H	7.2/2	7.2/2	7.2/2
	Placement DU/CU	ACO/RCO	MBS/ACO	ACO/RCO

**Table 7:** Summary of access optical connection traffic prediction.

	Benchmark		Context		Reduction [%]	
MSE	avg	max	avg	max	avg	max
Clustered	0.19	5.03	0.14	0.38	26%	92%
Distributed	0.26	5.43	0.17	0.84	35%	85%
Rel. Error	avg	max	avg	max	avg	max
Clustered	0.018	0.913	0.008	0.063	55%	93%
Distributed	0.019	0.551	0.012	0.059	37%	89%

## Data Availability

Data contained within the article.
